# Presence of Anticardiolipin Antibodies in Patients with Dementia: A Systematic Review and Meta-Analysis

**DOI:** 10.3389/fnagi.2017.00250

**Published:** 2017-08-02

**Authors:** Md. Asiful Islam, Fahmida Alam, Mohammad Amjad Kamal, Siew Hua Gan, Teguh Haryo Sasongko, Kah Keng Wong

**Affiliations:** ^1^Human Genome Centre, School of Medical Sciences, Universiti Sains Malaysia Kubang Kerian, Malaysia; ^2^King Fahd Medical Research Center, King Abdulaziz University Jeddah, Saudi Arabia; ^3^Enzymoics, Hebersham NSW, Australia; ^4^Novel Global Community Educational Foundation, Hebersham NSW, Australia; ^5^Division of Human Biology, School of Medicine, International Medical University Bukit Jalil, Malaysia; ^6^Department of Immunology, School of Medical Sciences, Universiti Sains Malaysia Kubang Kerian, Malaysia

**Keywords:** dementia, Alzheimer’s disease, antiphospholipid antibodies, anticardiolipin antibodies, systematic review, meta-analysis

## Abstract

Growing evidences are supporting towards the involvement of antiphospholipid antibodies [aPLs e.g., lupus anticoagulant (LA), anticardiolipin (aCL) and anti-β2-glycoprotein I (anti-β2-GPI) antibodies] in various neurological manifestations including migraine, epilepsy and dementia in the presence or absence of autoimmune diseases such as antiphospholipid syndrome or systemic lupus erythematosus. The aim of this systematic review and meta-analysis was to assess the presence of aPLs in dementia patients without a diagnosis of any autoimmune disease. Electronic databases (e.g., PubMed, Web of Science, Scopus, ScienceDirect and Google Scholar) were searched without any year or language restrictions and based on the inclusion criteria, nine prospective case-control studies assessing only aCL were included involving 372 dementia patients and 337 healthy controls. No studies were found to assess the presence of both LA or anti-β2-GPI. The study-specific odds ratios (ORs) and 95% confidence intervals (CIs) were calculated using random-effects model. We observed the prevalence of aCL in dementia was higher (32.80%) than that of controls (9.50%) e.g., 3.45 times higher risk of presenting with dementia than the controls, and significant presence of aCL antibodies was detected in dementia patients compared to controls (OR: 4.94, 95% CI: 2.66 – 9.16, *p* < 0.00001; *I^2^* = 32%, *p* = 0.16). Publication bias was not observed from Egger’s (*p* = 0.081) and Begg’s tests (*p* = 0.180). Based on the study quality assessment using modified Newcastle–Ottawa Scale for case-control studies, seven of nine studies were of high methodological quality scoring ≥ 7 (median value). In summary, aCL antibodies were significantly present in dementia patients suggesting that aCL antibodies are generated due to the autoimmune-derived effects of dementia or there might be a potential causative role of this autoantibody in dementia pathogenesis.

## Introduction

Dementia is a clinical syndrome that encompasses a set of neurologic symptoms involving difficulties in memory, speaking, problem solving, and thinking abilities, leading to the impairments of personal and social life (Román, 2003; Burns and Iliffe, 2009). It is most common in elderly people where advanced age being the strongest risk factor. A prevalence of 7.1% among the aged population (>65 years old) has been reported (Prince et al., 2014), and the number of people with dementia worldwide is estimated at 47 million and is projected to increase over 131 million by 2050 (Prince et al., 2016). Worldwide, the total number of new cases of dementia each year amounts to approximately 7.7 million, indicating one new case every 4.1 s (Prince et al., 2015).

Among several types of dementia, Alzheimer’s disease (AD) and vascular dementia (VD) are most commonly observed (Dening and Babu Sandilyan, 2015; Robinson et al., 2015). AD accounts for 60% whereas VD accounts for almost 30% of the prevalence (Kalaria et al., 2008). In AD, neurodegeneration occurs due to abnormal extracellular deposition of insoluble plaques consisting of Aβ peptides and intraneuronal aggregates of twisted fibers consisting of tau proteins (Dening and Babu Sandilyan, 2015). VD occurs when blood circulation to the brain is compromised due to arterial disease resulting in reduced neuronal function and eventually neurons cell death (Dening and Babu Sandilyan, 2015). In AD patients, the synthesis of intra-blood-brain barrier (BBB) IgG was observed which indicates an involvement of immune-mediated mechanisms in the pathogenesis of AD (Blennow et al., 1990).

In previous years, researches have been conducted on autoimmune diseases including antiphospholipid syndrome (APS) which may have links with the risk of dementia development (Gomez-Puerta et al., 2005; Lin et al., 2016). A recent study conducted on 1.8 million hospital cases reported that patients with autoimmune disorders including APS and systemic lupus erythematosus (SLE) were 20% more likely to develop dementia (Wotton and Goldacre, 2017), suggesting an autoimmune-mediated pathogenesis of dementia. In APS, presence of antiphospholipid antibodies (aPLs) (autoantibodies which react against anionic phospholipids and proteins on plasma membranes) namely anticardiolipin (aCL) antibody, anti-β2-glycoprotein I (β2GPI) antibody and lupus anticoagulant (LA) are found persistently in high titers (Miyakis et al., 2006; Giannakopoulos and Krilis, 2013). Presence of aPLs in high titers was also observed in APS patients suffering from different neurologic disorders including dementia (Islam et al., 2016, 2017a). Dementia has been observed in up to 56% APS patients (Chapman et al., 2002; Gomez-Puerta et al., 2005), and a study on non-SLE patients with neurological symptoms showed that over 50% of the patients with high levels of aPLs developed dementia (Inzelberg et al., 1992). Furthermore, aPLs are associated with impaired cognitive function (Schmidt et al., 1995) and the frequency of cognitive dysfunction is high ranging between 19 and 40% in aPLs-positive asymptomatic patients (Jacobson et al., 1999; Kozora et al., 2013).

The pathogenesis of aPL-mediated dementia in APS is not entirely understood. Suggested mechanisms include aPLs-induced BBB disruption (Katzav et al., 2010), aPLs-related microvascular thrombosis (Asherson et al., 1987; Denburg et al., 1997), or a direct effect of aPLs on brain tissues (Appenzeller et al., 2012). Thrombotic events triggered by aPL might contribute to the multiple cerebral thrombotic symptoms and greater aggression to the brain (de Godoy et al., 2000). Besides thrombotic effects, inflammatory and immune effects may contribute to the development of cognitive dysfunction in the presence of aPLs (Katzav et al., 2011).

To date, the association of aPLs in patients with dementia remains inconclusive based on the primary studies conducted on small number of dementia subjects. Certain studies have shown that aCL was significantly (*p* < 0.05) present in dementia patients versus healthy controls, 27% vs. 0% (Juby and Davis, 1998) or 28% vs. 3% (Tan et al., 2001). However, other studies did not report such significant association of aCL positivity in dementia versus healthy subjects, 29% vs. 26.4% (de Godoy et al., 2012). Thus, a systematic review and meta-analysis on all the primary studies was conducted to bring together all evidences in this topic and synthesize a conclusive information about the presence of aPLs in dementia patients. In addition, subgroup analyses were performed to evaluate the presence of aCL in different types of dementia, distinct age ranges and patients in different geographical continents.

## Materials and Methods

To conduct this meta-analysis, we followed the guidelines published by the Meta-analysis of Observational Studies in Epidemiology (MOOSE) group (Supplementary Table S1) (Stroup et al., 2000).

### Study Selection Criteria

Studies were included if: (1) Study design was prospective case-control; (2) The aim of the study was to evaluate the existence of aPLs (LA, aCL, and anti-β2-GPI antibodies) in patients with dementia; (3) Dementia subjects were of any age, sex or race without any underlying autoimmune disorders such as APS or SLE.

### Literature Search

A systematic literature search using ‘Advanced’ and ‘Expert’ search strategies of PubMed, Web of Science, Scopus, Science Direct, and Google Scholar databases was independently conducted by two researchers (MAI and FA), and the shortlisted studies were independently verified by KKW. There were no search year or language restrictions. Review articles, case reports, clinical trials, editorials, letters, and comments were excluded. Studies were also excluded if overlapping of identical study subjects was observed with other included studies from similar research group. To ensure that there were no potential papers overlooked, we examined the reference list of selected studies and reviewed publications that had cited the selected studies (via Google Scholar). The electronic search included both Medical Subject Heading (MeSH) in addition of appropriate keywords and combined with the Boolean operators (‘AND’ and ‘OR’). The following search terms were used: (antiphospholipid antibody *OR* antiphospholipid antibodies *OR* anticardiolipin antibody *OR* anticardiolipin antibodies *OR* lupus anticoagulant *OR* β2GPI *OR* β2-GPI *OR* β2glycoprotein *OR* β2-glycoprotein) *AND* (dementia *OR* Alzheimer *OR* Alzheimer’s). The final systematic search was conducted on 12th March 2017 (Supplementary Table S2).

### Data Extraction, Management and Quality Assessment

Two researchers (MAI and FA) independently extracted the following data from each of the selected studies: first author and year (study ID), study design, country, number of dementia patients and controls (number of female patients and controls), types of dementia, mean age of dementia patients and controls, types and isotypes of tested aPLs, dementia diagnostic criteria, aPLs measurement techniques and cut-off values. To resolve any discrepancies such as unclear or missing data presentation, all authors took part in the discussion. If not resolved, we then contacted either the corresponding or the first author of the respective study for further clarifications. By using a modified version of the Newcastle–Ottawa Scale (NOS) (Islam et al., 2017b), quality of each of the selected studies was assessed; studies scoring above the median NOS value were considered as high quality (low risk of bias) and those scoring below the median value were considered as low quality (high risk of bias) (Wu et al., 2016).

### Exploration of Heterogeneity and Publication Bias

Heterogeneity across studies was tested based on *I*^2^ statistics which indicates the percentage of variance attributable to study heterogeneity. Studies with *I*^2^< 40%, *I*^2^ = 40–75% or *I*^2^ > 75% was considered to have low, moderate or high heterogeneity (Harris et al., 2015). Three independent subgroup analyses were conducted as follows: (1) VD vs. dementia of the Alzheimer’s type (DAT); (2) Age ranged from 60 to 70 years vs. above 70 years old; (3) Subjects from Asia and Europe vs. North and South America. Additionally, L’Abbé plot was generated for the visual inspection of heterogeneity by using RStudio (version 1.0.136) software (metafor package, version 1.9-9) (Viechtbauer, 2010).

Publication bias was visually assessed by using funnel plots. Moreover, Egger’s regression test (Egger et al., 1997) and Begg’s test (Begg and Mazumdar, 1994) was conducted to further assess publication bias with random-effects model. Publication bias was considered significant if *p* < 0.05. Funnel plot was illustrated with the metafor package, version 1.9-9 (Viechtbauer, 2010).

### Statistical Analyses of Meta-Analysis

Random-effects model was used to conduct this meta-analysis. Odds ratio (OR) was used to evaluate the comorbid association of the presence of aPLs in dementia patients compared to controls where *p* < 0.05 was considered significant. RevMan (Cochrane Collaboration, software version 5.3.5) (The Cochrane Collaboration, 2014) was used to generate the forest plot.

## Results

### Study Selection

**FIGURE 1:**
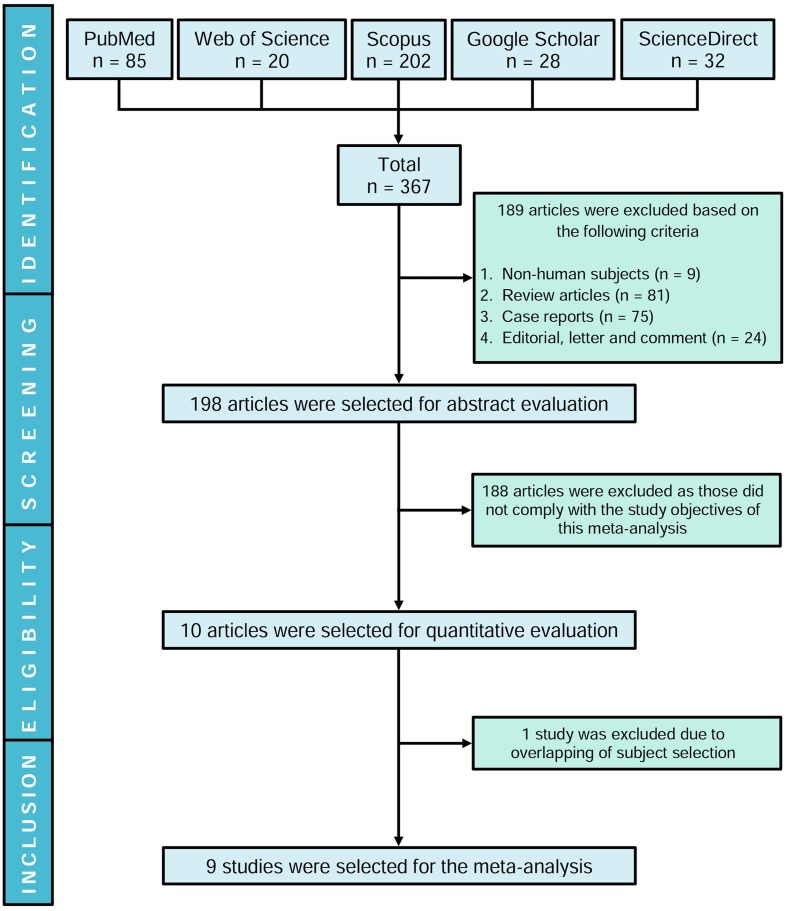
Flow chart illustrating the study selection process in the meta-analysis.

Our initial search yielded 367 articles where 189 studies were excluded and the remaining 198 articles were evaluated based on title and abstract. Ten studies were shortlisted following the inclusion criteria, and one article (Juby et al., 1995) was excluded due to overlapping of identical study subjects with another eligible study (Juby and Davis, 1998). Therefore, nine studies were included in this meta-analysis (**Figure 1**). Although initially the aim was to evaluate the presence of aPLs in dementia patients, based on our search strategies and systematic review, we could not find studies evaluating LA or anti-β2GPI except for aCL.

### Study Characteristics and Quality Assessment

Among the included studies, four were from China (Tan et al., 2001; Zhao and Tan, 2004; Zeng et al., 2006; Qian et al., 2015), two were from Brazil (de Godoy et al., 2005, 2012), and the remaining three studies were from Israel (Mosek et al., 2000), Canada (Juby and Davis, 1998), and United States (Lopez et al., 1992). Across the nine studies, there were 709 subjects (dementia patients: *n* = 372; controls: *n* = 337) in total. All of the studies were designed as case-control and evaluated the presence of aCL as the only aPLs in dementia patients compared to controls. In particular, six of the studies were on VD (Lopez et al., 1992; Tan et al., 2001; Zhao and Tan, 2004; de Godoy et al., 2005; Zeng et al., 2006; Qian et al., 2015) and two on DAT (Juby and Davis, 1998; de Godoy et al., 2012) and one study with both VD and DAT patients (Mosek et al., 2000). The age range of the dementia patients and controls was 65–80.5 and 50.1–78.3 years, respectively. **Table 1** summarizes the major characteristics of the included studies.

**Table 1:** Major characteristics of the case-control studies mentioned in the meta-analysis.

No.	Study ID	Country	Number of dementia patients (number of female)	Types of dementia (n)	Mean age of dementia patients (years)	Number of controls (number of female)	Mean age of controls (years)	Types of tested aPLs (isotype)	Dementia diagnostic criteria	aCL measurement (test; cut-off)
1	Qian 2015	China	32 (17)	VD (32)	68.7	31 (15)	67.2	aCL (IgG)	DSM-III	ELISA; NR
2	de Godoy 2012	Brazil	31 (23)	DAT (31)	71.2	34 (25)	68.0	aCL (IgG and IgM)	NINCDS-ADRDA	ELISA; NR
3	Zeng 2006	China	75 (31)	VD (75)	65.5	30 (15)	50.1	aCL (IgG and IgM)	NR	ELISA; IgG > 20 GPL, IgM > 20 MPL
4	de Godoy 2005	Brazil	30 (13)	VD (30)	67.2	34 (25)	68.0	aCL (IgG and IgM)	NINCDS-ADRDA	ELISA; IgG > 10 GPL, IgM > 7 MPL
5	Zhao 2004	China	39 (15)	BD (39)	80.5	36 (14)	78.3	aCL (NR)	NR	ELISA; NR
6	Tan 2001	China	31 (11)	VD (31)	66.8	30 (12)	66.2	aCL (IgG)	DSM-I	ELISA; IgG > 20 U/mL
7	Mosek 2000	Israel	87 (48)	DAT (68)	74.0	69 (40)	78.0	aCL (IgG)	DSM-IV	ELISA; IgG > 20 GPL
				MD (3)						
				VD (16)						
8	Juby 1998	Canada	37 (NR)	DAT (26)	>65.0	63 (NR)	>65.0	aCL (INR)	NR	ELISA; NR
				VD (11)						
9	Lopez 1992	United States	10 (3)	VD (10)	72.4	10 (6)	72.1	aCL (IgG)	NINCDS-ADRDA	ELISA; NR


*VD, vascular dementia; BD, Binswanger’s disease; DAT, dementia of the Alzheimer’s type; MD, mixed dementia; aPLs, antiphospholipid antibodies; NINCDS-ADRDA, National Institute of Neurological and Communicative Disorders and Stroke and the Alzheimer’s Disease and Related Disorders Association; DSM-IV, Diagnostic and Statistical Manual of Mental Disorders IV; NR, not reported; ELISA, enzyme-linked immunosorbent assay; IgG, immunoglobulin G; IgM, immunoglobulin M; GPL, immunoglobulin G phospholipid; MPL, Immunoglobulin M phospholipid.*

Quality assessment of the included studies by using NOS for case-control studies is shown in **Table 2**. The median score of NOS was 7. Among the nine studies, seven studies were of high quality (low risk of bias) scoring ≥ 7 (Lopez et al., 1992; Juby and Davis, 1998; Mosek et al., 2000; Tan et al., 2001; de Godoy et al., 2005, 2012; Qian et al., 2015) and two studies were of low quality (high risk of bias) scoring < 7 (Zhao and Tan, 2004; Zeng et al., 2006).

**Table 2:** Risk of bias assessment of the included studies according to the modified Newcastle-Ottawa Scale (NOS).

Quality assessment	Qian 2015	de Godoy 2012	Zeng 2006	de Godoy 2005	Zhao 2004	Tan 2001	Mosek 2000	Juby 1998	Lopez 1992
**Selection**					
(1) Is the case definition adequate?	★	★	○	★	○	★	★	○	★
(2) Representativeness of the cases	★	★	★	★	★	★	★	★	★
(3) Selection of controls	○	★	○	★	○	○	★	★	○
(4) Definition of controls	★	○	★	○	★	○	★	★	★
**Comparability**					
(5) Study controls for the most important factor	★	★	○	★	★	★	★	★	★
(6) Study controls for the second important factor	★	★	○	★	○	★	★	★	○
**Exposure**									
(7) Was the measurement method of aPLs described?	★	★	★	★	★	★	★	★	★
(8) Were the methods of measurements same for cases and controls (e.g., ELISA)?	★	★	★	★	★	★	★	★	★
(9) Non-response rate	★	★	★	★	★	★	★	★	★


### Assessment of aCL Presence by Meta-Analysis

**FIGURE 2:**
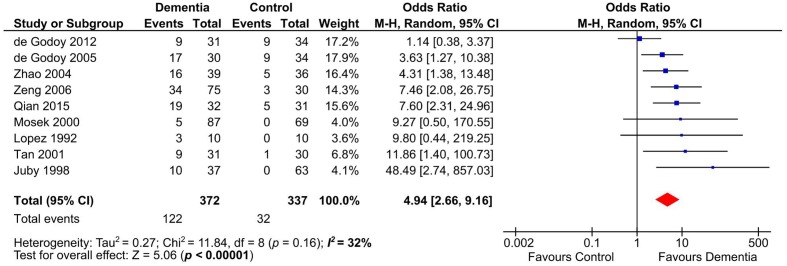
Forest plot representing the presence of anticardiolipin (aCL) antibodies in dementia patients compared to controls.

Presence of aCL in dementia patients was highly significant compared to controls (OR: 4.94, 95% CI: 2.66–9.16, *p* < 0.00001; *I*^2^ = 32%, *p* = 0.16) (**Figure 2**). aCL was present in 32.80% of dementia patients and 9.50% in controls corresponding to 3.45 times higher probability to present with dementia than controls.

### Subgroup Analyses on VD and DAT

**FIGURE 3:**
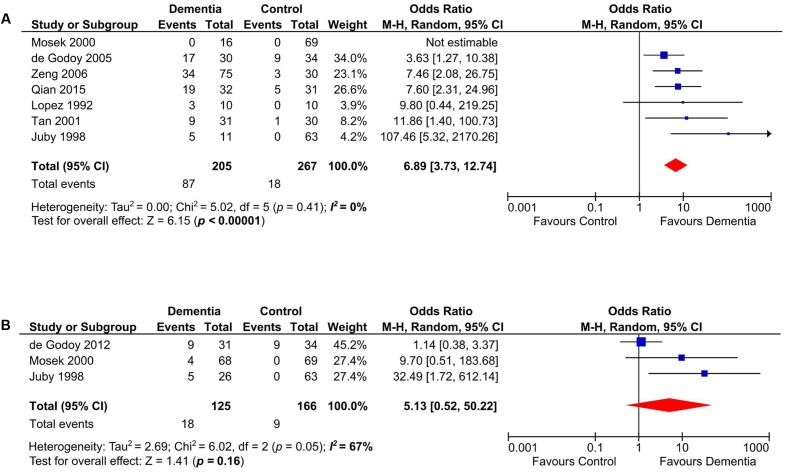
Subgroup analysis of aCL in patients with vascular dementia (VD) **(A)** and DAT **(B)**.

Our meta-analysis on studies that evaluated the presence of aCL in patients with VD (*n* = 472) (Lopez et al., 1992; Juby and Davis, 1998; Mosek et al., 2000; Tan et al., 2001; de Godoy et al., 2005; Zeng et al., 2006; Qian et al., 2015) indicated that aCL was significantly present in VD patients as compared to healthy controls (OR: 6.89, 95% CI: 3.73–12.74, *p* < 0.00001; *I*^2^ = 0%, *p* = 0.41) (**Figure 3A**). For studies that measured aCL antibodies in DAT patients (*n* = 291) (Juby and Davis, 1998; Mosek et al., 2000; de Godoy et al., 2012), aCL was not significantly associated with DAT (OR: 5.13, 95% CI: 0.52–50.22, *p* = 0.16; *I*^2^ = 67%, *p* = 0.05) (**Figure 3B**).

### Subgroup Analyses on Different Age Ranges

**FIGURE 4:**
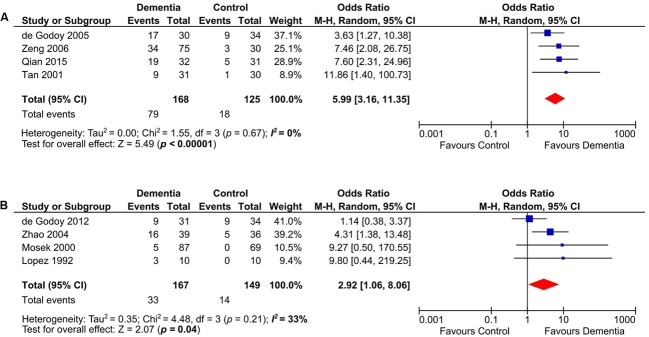
Subgroup analysis of aCL in demetia patients with age range of 60–70 years **(A)** and above 70 years old **(B)**.

Anticardiolipin was significantly present in dementia subjects of age groups between 60 and 70 years old (*n* = 293) (Tan et al., 2001; de Godoy et al., 2005; Zeng et al., 2006; Qian et al., 2015) (OR: 5.99, 95% CI: 3.16–11.35, *p* < 0.00001; *I*^2^ = 0%, *p* = 0.67) (**Figure 4A**) and those more than 70 years old (*n* = 316) (Lopez et al., 1992; Mosek et al., 2000; Zhao and Tan, 2004; de Godoy et al., 2012) (OR: 2.92, 95% CI: 1.06–8.06, *p* = 0.04; *I*^2^ = 33%, *p* = 0.21) (**Figure 4B**).

### Subgroup Analyses on Patients in Different Continents

**FIGURE 5:**
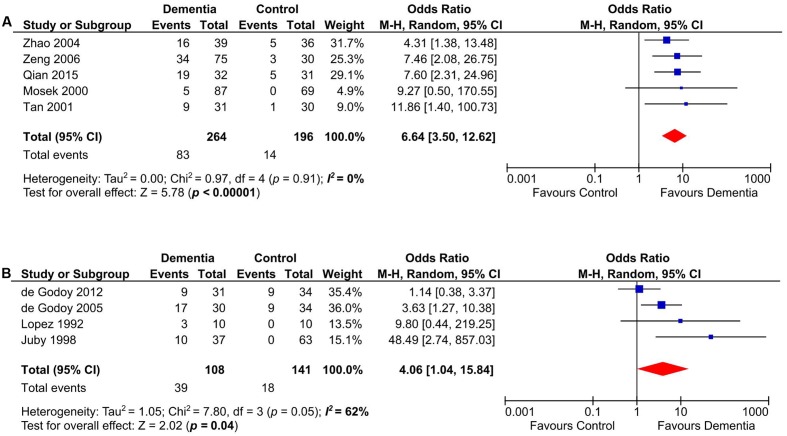
Subgroup analysis of aCL in Asian and European **(A)**, North and South American **(B)** dementia populations.

Anticardiolipin antibody was significantly present in Asian and European dementia patients (*n* = 460) (Mosek et al., 2000; Tan et al., 2001; Zhao and Tan, 2004; Zeng et al., 2006; Qian et al., 2015) (OR: 6.64, 95% CI: 3.50–12.62, *p* < 0.00001; *I*^2^ = 0%, *p* = 0.91) (**Figure 5A**), as well as in North and South American dementia subjects (*n* = 249) (Lopez et al., 1992; Juby and Davis, 1998; de Godoy et al., 2005, 2012) (OR: 4.06, 95% CI: 1.04–15.84, *p* = 0.04; *I*^2^ = 62%, *p* = 0.05) (**Figure 5B**).

### Heterogeneity and Publication Bias

**FIGURE 6:**
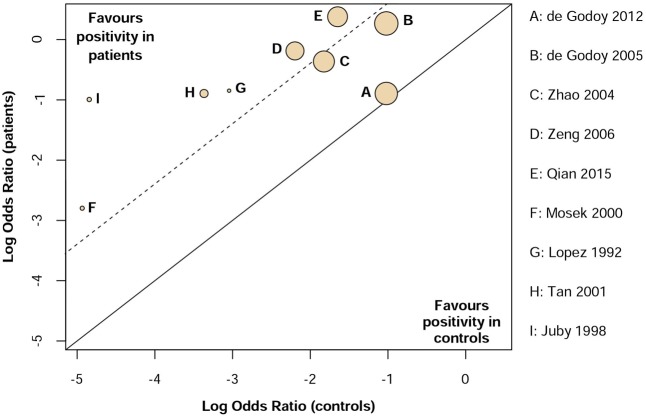
L’Abbé plot suggests no substantial heterogeneity for the assessment of aCL antibodies.

Low heterogeneity was observed (*I*^2^ = 32%) in assessing aCL in dementia patients compared to controls. Additionally, visual inspection of L’Abbé plot (**Figure 6**) demonstrated no substantial heterogeneity.

**FIGURE 7:**
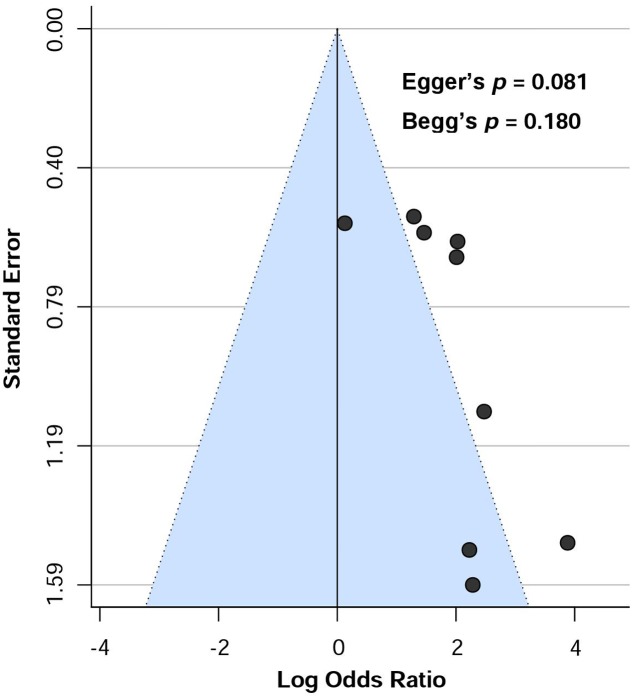
Funnel plot showing the risk of publication bias assessing the presence of aCL antibodies in dementia patients.

Visual assessment of funnel plot (**Figure 7**) showed that the studies were distributed asymmetrically, suggesting the presence of some publication bias. Begg’s test was not significant (*p* = 0.180), however, there was a trend toward significance for Egger’s regression (*p* = 0.081).

## Discussion

In this study, based on the meta-analysis of nine shortlisted studies (372 dementia patients and 337 healthy controls), we validated the fact that aCL antibodies were significantly present in dementia patients as compared to healthy subjects, thus resolving previous conflicting reports on their associations. Our observation based on the meta-analysis on case-control studies was also supported by some cohort studies demonstrating the association of aCL positivity with cognitive decline and impaired motor function as follows: (1) Jacobson et al. (1999) reported that the frequency of impaired neuropsychologic performance was significantly higher among young individuals with aPLs (*n* = 27) as compared with controls (*p* < 0.01). (2) Primary data of a longitudinal study demonstrated that aCL was positive up to 19% of subjects with impaired cognitive and motor function (Arvanitakis et al., 2012); (3) Another cohort study on normal population (*n* = 1895) without neurological disease including dementia indicated that aCL-positive subjects performed worst on the Mini Mental State Examination (MMSE) cognitive scale, suggesting an aCL-mediated mechanism in cognitive decline (Homayoon et al., 2014).

In addition, our subgroup analyses showed that aCL was significantly present in patients with VD but not DAT. aCL has been observed to be associated with stroke development (Levine et al., 1987; Muir et al., 1994; Janardhan et al., 2004). It has been hypothesized that aCL-induced thrombotic events may contribute to multiple cerebral thrombotic symptoms and exert greater aggression to the brain (de Godoy et al., 2000). aCL antibodies may exert VD via vascular events similar with that seen in aCL-associated strokes (Tan et al., 2001).

Although dementia has been observed to comorbid with aPLs, the causative role of aPLs in dementia is still inconclusive. The BBB is an interface comprising of endothelial cells, astrocyte end-feet and pericytes, separating the brain involving neurons, blood vessels and glial cells from the circulatory system (Ballabh et al., 2004; Capani et al., 2016). BBB protects the central nervous system (CNS) by blocking the entry of harmful substances while allowing the transport of essential molecules (Lee et al., 2016). Interestingly, aCL antibodies have shown a direct binding affinity towards astrocytes in an *in vivo* study (Sun et al., 1992). aCL could inhibit the proliferation of astrocytes that ultimately distort the structure and function of BBB in addition of eliciting thrombus formation in the brain’s blood vessels (Yu et al., 1991; Sun et al., 1992). Direct binding of aPLs to brain tissues (Kent et al., 1997; Caronti et al., 1998a,b; Kent et al., 2000) after BBB disruption might be a potential pathogenic mechanism of dementia development.

aPLs mainly react with phospholipids, phospholipid-protein complexes, and phospholipid-binding proteins (Fischer et al., 2007; Misasi et al., 2015). Brain tissues comprising of gray and white-matter contain high proportion of phospholipids (especially on brain cell membranes) such as phosphatidylcholine, phosphatidylserine, phosphatidylinositol, and sphingomyelin (Hamberger and Svennerholm, 1971; Kwee and Nakada, 1988; Calderon et al., 1995) which may become the target of aCL antibodies. In an *in vivo* study, aCL antibodies were found to bind with only brain tissues when compared with liver tissues (Sun et al., 1992). In addition, through a damaged BBB, aCL antibodies were also found to diffuse from blood circulation to CNS as aCL antibodies were simultaneously found in the cerebrospinal fluids of different neurologic disorders such as multiple sclerosis, neurosyphilis and Guillain–Barré syndrome (Harris et al., 1985). Thus, there might exist a selective mechanism of aCL antibody binding with phospholipids of brain tissues, contributing to dementia pathogenesis.

Past studies have shown that aPL-positive subjects for more than 20 years had higher risk of developing dementia (Mosek et al., 2000; Chapman et al., 2002). In transgenic animal model of AD, prolonged exposure of aPLs in the brain was found to generate AD-like pathology including accumulation of amyloid peptides, formation of mature amyloid plaque as well as development of behavioral and cognitive changes (Katzav et al., 2011). The researchers proposed that BBB break down might occur via inflammation, coagulation and direct antibody binding such as aPLs (Katzav et al., 2011).

In patients with VD, cognitive impairments occur due to cerebrovascular disease and ischemic or hemorrhagic brain injury (Iemolo et al., 2009). Increasing evidences of BBB dysfunction have been observed in stroke patients and cerebrovascular incidents are believed to play significant roles in VD development (Ueno et al., 2015; van de Haar et al., 2015). On the other hand, altered BBB was also observed in VD without brain infarcts, suggesting infarction-beyond pathogenesis of VD via BBB disruption (Wallin et al., 1990). Cerebrovascular events have been observed in patients exhibiting aCL antibodies and thought to contribute in the pathogenesis via triggering thrombotic events (Kushner, 1990; de Godoy et al., 2000; Brey et al., 2002). Chapman et al. (1999) reported that IgG aCL antibodies could disrupt neuronal function via permeabilization and depolarization of brain synaptoneurosomes by direct action on nerve terminals. Therefore, subsequent chronic permeabilization could lead to irreversible damage and neuronal loss which might explain our findings of significant presence of aCL in patients with dementia. Therefore, in terms of VD, aCL antibodies might have indirect pathogenic contributions via either developing cerebrovascular events or direct immune-mediated mechanisms.

Blood-brain barrier dysfunction was reported in a group of demented patients (AD = 56; VD = 29) with white-matter changes without evidences of stroke (Wallin et al., 2000). This study concludes that BBB dysfunction might be linked with vasculature and tissue damage. Interestingly, a significant association was observed (*p* < 0.05) between the presence of aCL antibodies and cerebral damage in white-matter of neuropsychiatric SLE patients (Steens et al., 2006). Another study reported that reduced white-matter volume was associated with the presence of aPLs in SLE patients with cognitive impairment (Appenzeller et al., 2007). Therefore, besides BBB, white-matter region could be a potential target of aCL antibodies in the pathogenesis of dementia.

In both AD and VD, oxidative stress is an established phenomenon to be involved in the pathogenesis (Cervellati et al., 2014; Luca et al., 2015; Alam et al., 2016; Islam et al., 2017c). In an experimental mouse model, aCL antibodies were significantly associated with decreased paraoxonase activity and reduced nitric oxide levels (Alves et al., 2005), which suggests the involvement of aCL in inducing oxidative stress.

Several limitations should be noted in this meta-analysis. Firstly, the number of included studies (*n* = 9) in the meta-analysis was relatively low. However, aCL was significantly associated with dementia patients regardless of age ranges (60–70 vs. above 70 years old) nor patients from different geographical continents (Asian and European vs. North and South Americans), suggesting the reproducibility of aCL-dementia association across different age groups and nationalities. Secondly, the cut-off values of aCL antibody positivity were different from one study to another. Thirdly, the diagnostic criteria followed to confirm dementia varied across the studies. Finally, although neither Begg’s nor Egger’s tests showed significant publication bias, visual inspection of the funnel plot demonstrated asymmetrical distribution of included studies showing a trend towards publication bias. This discrepancy was possibly due assessment of heterogeneity using lower number of studies (<10).

Although a few case-reports and cohort studies reported the presence (Inzelberg et al., 1992; Kurita et al., 1994; Van Horn et al., 1996; Ciubotaru et al., 2002) or absence (Friedman, 2011; De Maeseneire et al., 2014) of anti-β2-GPI and LA in dementia patients, to the best of our knowledge, no case-control studies assessing anti-β2-GPI and LA in dementia patients have been conducted. In addition, the role of aCL antibodies in the pathogenic mechanisms of dementia remains unclear, and further research is required to establish the potential involvement of aCL antibodies in the pathogenesis of dementia.

## Conclusion

Anticardiolipin antibodies were significantly present in dementia patients compared to healthy controls, underscoring the potential to screen aCL-positive subjects for early symptoms of neurological impairment and dementia, as well as suggesting the important role of aCL antibodies in the pathogenesis of dementia.

## Author Contributions

MAI and KKW conceived and designed the study. MAI and FA searched the databases and KKW participated in the study selection process. MAI, FA and KKW analyzed and interpreted the data. MAI, FA and KKW drafted the manuscript. THS, SHG and MAK critically edited, reviewed and approved the final version of the submitted manuscript.

## Conflict of Interest Statement

The authors declare that the research was conducted in the absence of any commercial or financial relationships that could be construed as a potential conflict of interest.
