# Supplementary material for: Presence of Anticardiolipin Antibodies in Patients with Dementia: A Systematic Review and Meta-Analysis
Source: Front Aging Neurosci. 2017 Aug 2;9:250. doi: 10.3389/fnagi.2017.00250 (PMC5539075; doi:10.3389/fnagi.2017.00250)
Supplement: Supplementary file 1 [file Table_1.DOCX]

***Supplementary Material***

**Presence of anticardiolipin antibodies in patients with dementia: A systematic review and meta-analysis**

**Md. Asiful Islam*, Fahmida Alam, Mohammad Amjad Kamal, Siew Hua Gan, Teguh Haryo Sasongko, Kah Keng Wong***

***Correspondence**

**Corresponding Authors:** Md. Asiful Islam ([ayoncx70@yahoo.com](mailto:ayoncx70@yahoo.com)) & Kah Keng Wong ([kahkeng@usm.my](mailto:kahkeng@usm.my))

| **Supplementary Table S1: MOOSE Checklist** | | | |
| --- | --- | --- | --- |
| **Item No** | **Criteria** | **Sentences mentioned in the meta-analysis or appropriate explanations** |  |
| 1 | Problem definition | To date, the association of aPLs in patients with dementia remains inconclusive. Some studies reported significant association, whereas some studies found negative results. |  |
| 2 | Hypothesis statement | Besides thrombotic effects, inflammatory and immune effects may contribute to the development of cognitive dysfunction in the presence of aPLs |  |
| 3 | Description of study outcome(s) | To provide a more appropriate answer regarding the association of aPLs presence in patients with dementia. |  |
| 4 | Type of exposure or intervention used | Presence of aPLs [aCL (IgG, IgM and IgA), anti-β2-GPI antibodies (IgG, IgM and IgA) and LA] in both patients and controls. |  |
| 5 | Type of study designs used | Prospective case-control studies. |  |
| 6 | Study population | Dementia patients of any age, sex or race (without any underlying autoimmune diseases such as APS and SLE) were considered eligible. Controls without any underlying autoimmune (e.g., APS and SLE) or neurologic disorders diseases were considered eligible control participants. |  |
| 7 | Qualifications of searchers (e.g., librarians and investigators) | M.A.I. (Ph.D. Scholar, investigator), F.A. (Ph.D. Scholar, investigator), K.K.W. (Principal Investigator & University Lecturer). |  |
| 8 | Search strategy, including time period included in the synthesis and key words | The final systematic search was conducted on 12th March, 2017 without language and search year restrictions. |  |
| 9 | Effort to include all available studies, including contact with authors | We contacted either the corresponding or the first author of the respective study. |  |
| 10 | Databases and registries searched | PubMed, Web of Science, Scopus, ScienceDirect and Google Scholar. |  |
| 11 | Search software used, name and version, including special features used (e.g., explosion) | We did not employ a search software. EndNote was used to merge retrieved citations and eliminate duplications. |  |
| 12 | Use of hand searching (e.g., reference lists of obtained articles) | We used hand search strategy by checking the references listed in the selected articles and also checked the citation list of the selected articles from the Google Scholar. |  |
| 13 | List of citations located and those excluded, including justification | Details of the literature search process are outlined in the flow diagram (Figure 1). |  |
| 14 | Method of addressing articles published in languages other than English | We placed no restrictions on language. |  |
| 15 | Method of handling abstracts and unpublished studies | We did not consider unpublished studies to be included in the analysis. |  |
| 16 | Description of any contact with authors | For clarification, we contacted with the corresponding author of the two studies which we assumed that identical/similar subject cohort (both patients and controls) was used in both studies. |  |
| 17 | Description of relevance or appropriateness of studies assembled for assessing the hypothesis to be tested | Detailed inclusion and exclusion criteria were described under in *Study selection criteria* of “Methods” section. |  |
| 18 | Rationale for the selection and coding of data (e.g., sound clinical principles or convenience) | Selection of the studies was based on the inclusion and exclusion criteria. |  |
| 19 | Documentation of how data were classified and coded (e.g., multiple raters, blinding and interrater reliability) | Mentioned in the “Data extraction, management and quality assessment” section. |  |
| 20 | Assessment of confounding (e.g., comparability of cases and controls in studies where appropriate) | Three subgroup analyses were carried out (Figure 3, Figure 4 and Figure 5) |  |
| 21 | Assessment of study quality, including blinding of quality assessors, stratification or regression on possible predictors of study results | Quality assessment of each of the included studies was evaluated following a modified version of Newcastle-Ottawa Scale (NOS). |  |
| 22 | Assessment of heterogeneity | Begg’s, Egger’s tests and L'Abbé plots were used. |  |
| 23 | Description of statistical methods (e.g., complete description of fixed or random effects models, justification of whether the chosen models account for predictors of study results, dose-response models, or cumulative meta-analysis) in sufficient detail to be replicated | Described in the “Statistical analyses of meta-analysis” section. |  |
| 24 | Provision of appropriate tables and graphics | We included detailing the terms used for database search under “Supplementary Table S2” section, one flow chart (Figure 1), one forest plot (Figure 2), one L'Abbé plot (Figure 6), one funnel plot (Figure 7), one summary table (Table 1) and one risk of bias assessment table (Table 2). |  |
| 25 | Graphic summarizing individual study estimates and overall estimate | One forest plot (Figure 2). |  |
| 26 | Table giving descriptive information for each study included | Table 1 |  |
| 27 | Results of sensitivity testing (e.g., subgroup analysis) | Three subgroup analyses were carried out between VD and DAT (Figure 3); subjects age with 60 – 70 years and > 70 years (Figure 4); dementia population from Asia and Europe, North and South America (Figure 5). All the results were significant except DAT (*p* < 0.16). |  |
| 28 | Indication of statistical uncertainty of findings | 95% confidence intervals were presented with all summary estimates, I^2^ values and results of subgroup analyses. |  |
| 29 | Quantitative assessment of bias (e.g., publication bias) | Funnel plot (Figure 7). |  |
| 30 | Justification for exclusion (e.g., exclusion of non-English language citations) | Flow chart (Figure 1). |  |
| 31 | Assessment of quality of included studies | Table 2. |  |
| 32 | Consideration of alternative explanations for observed results | Discussed in the “Discussion” section. |  |
| 33 | Generalization of the conclusions (i.e., appropriate for the data presented and within the domain of the literature review) | Discussed at the end of the “Discussion” section. |  |
| 34 | Guidelines for future research | Discussed at the end of the “Discussion” section. |  |
| 35 | Disclosure of funding source | None declared. |  |
