# Supplementary material for: Presence of Anticardiolipin Antibodies in Patients with Dementia: A Systematic Review and Meta-Analysis
Source: Front Aging Neurosci. 2017 Aug 2;9:250. doi: 10.3389/fnagi.2017.00250 (PMC5539075; doi:10.3389/fnagi.2017.00250)
Supplement: Supplementary file 2 [file Table_2.DOCX]

***Supplementary Material***

**Presence of anticardiolipin antibodies in patients with dementia: A systematic review and meta-analysis**

**Md. Asiful Islam^*^, Fahmida Alam, Mohammad Amjad Kamal, Siew Hua Gan, Teguh Haryo Sasongko, Kah Keng Wong***

***Correspondence**

**Corresponding Authors:** Md. Asiful Islam ([ayoncx70@yahoo.com](mailto:ayoncx70@yahoo.com)) & Kah Keng Wong ([kahkeng@usm.my](mailto:kahkeng@usm.my))

| **Supplementary Table S2: Detailed Search Strategy** | |
| --- | --- |
| **Databases** | **Search Strategy** |
| **PubMed** | ((((((((((antiphospholipid antibody[Title/Abstract]) OR antiphospholipid antibodies[Title/Abstract]) OR anticardiolipin antibody[Title/Abstract]) OR anticardiolipin antibodies[Title/Abstract]) OR lupus anticoagulant[Title/Abstract]) OR β2GPI[Title/Abstract]) OR β2-GPI[Title/Abstract]) OR β2glycoprotein[Title/Abstract]) OR β2-glycoprotein[Title/Abstract])) AND (((dementia[Title/Abstract]) OR Alzheimer disease[Title/Abstract]) OR Alzheimer's disease[Title/Abstract]) |
| **Web of Science** | (TI=antiphospholipid antibody OR TI=antiphospholipid antibodies OR TI=anticardiolipin antibody OR TI=anticardiolipin antibodies OR TI=lupus anticoagulant OR TI=β2GPI OR TI=β2-GPI OR TI=β2glycoprotein OR TI=β2-glycoprotein) AND (TI=dementia OR TI=Alzheimer disease OR TI=Alzheimer's disease) |
| **Scopus** | (TITLE-ABS-KEY (antiphospholipid AND antibody) OR TITLE-ABS-KEY (antiphospholipid AND antibodies) OR TITLE-ABS-KEY (anticardiolipin AND antibody) OR TITLE-ABS-KEY (anticardiolipin AND antibodies) OR TITLE-ABS-KEY (lupus AND anticoagulant) OR TITLE-ABS-KEY (β2GPI) OR TITLE-ABS-KEY (β2-GPI) OR TITLE-ABS-KEY (β2glycoprotein) OR TITLE-ABS-KEY (β2-glycoprotein) AND TITLE-ABS-KEY (dementia) OR TITLE-ABS-KEY (Alzheimer AND disease) OR TITLE-ABS-KEY (Alzheimer's AND disease)) |
| **Google Scholar** | allintitle: “antiphospholipid antibody” “dementia”; allintitle: "antiphospholipid antibodies" "dementia"; allintitle: "anticardiolipin antibody" "dementia"; allintitle: "anticardiolipin antibodies" "dementia"; allintitle: "lupus anticoagulant" "dementia"; allintitle: " β2-glycoprotein" "dementia"; allintitle: " β2-glycoprotein" "dementia"; allintitle: “antiphospholipid antibody” “Alzheimer’s”; allintitle: "antiphospholipid antibodies" "Alzheimer’s"; allintitle: "anticardiolipin antibody" "Alzheimer’s"; allintitle: "anticardiolipin antibodies" "Alzheimer’s"; allintitle: "lupus anticoagulant" "Alzheimer’s"; allintitle: " β2-glycoprotein" "Alzheimer’s"; allintitle: " β2-glycoprotein" "Alzheimer’s" |
| **ScienceDirect** | TITLE-ABSTR-KEY(antiphospholipid*) or TITLE-ABSTR-KEY(anticardiolipin*) or TITLE-ABSTR-KEY(lupus anticoagulant) or TITLE-ABSTR-KEY(β2GP) or TITLE-ABSTR-KEY(β2-GP) or TITLE-ABSTR-KEY(β2glycoprotein) or TITLE-ABSTR-KEY(β2-glycoprotein) and TITLE-ABSTR-KEY(dementia*) or TITLE-ABSTR-KEY(Alzheimer*) |
